# The role of ear stone size in hair cell acoustic sensory transduction

**DOI:** 10.1038/srep02114

**Published:** 2013-07-02

**Authors:** Maya Inoue, Masashi Tanimoto, Yoichi Oda

**Affiliations:** 1 Division of Biological Science, Graduate School of Science, Nagoya University, Japan; 2 Research Fellow DC of the Japan Society for the Promotion of Science, Japan

## Abstract

Hearing and bodily balance are different sensations initiated by a common mechanism. Both sound- and head movement-dependent mechanical displacement are converted into electrical signals by the sensory hair cells. The saccule and utricle inner ear organs, in combination with their central projections to the hindbrain, are considered essential in fish for separating auditory and vestibular stimuli. Here, we established an *in vivo* method in larval zebrafish to manipulate otolith growth. We found that the saccule containing a large otolith is necessary to detect sound, whereas the utricle containing a small otolith is not sufficient. Otolith removal and relocation altered otolith growth such that utricles with experimentally enlarged otoliths acquired the sense of sound. These results show that otolith biomineralization occurs in a region-specific manner, and suggest that regulation of otolith size in the larval zebrafish ear is crucial to differentially sense auditory and vestibular information.

In vertebrates, sound and head movement are detected by the inner ear. In the mammalian inner ear, the cochlea transduces sound stimuli, whereas the otolith organs and semicircular canals transduce linear and angular acceleration, respectively. Although hearing and balance are distinct sensations, their transduction occurs through a common mechanism: hair cells convert auditory or vestibular stimuli into electrical signals^1^. The mechanical coupling between hair cells and extracellular structures in the ear provides a mechanism through which auditory and vestibular stimuli can be differentiated and sensed independently. Nevertheless, it is uncertain to what extent the changes in the extracellular structures can affect these 2 senses.

The cochlea is absent in fish; instead, their inner ear contains 3 otolith organs that receive both auditory and vestibular stimuli^2^,^3^,^4^. The otolith organs contain macular sensory hair cells that are coupled with an otolith, a biomineralized ear stone composed of calcium carbonate and proteins. The otolith acts as an inertial mass, and sound- and head movement-evoked acceleration produces relative displacement between the otolith and the coupled hair cells due to the difference in their inertia. This displacement mechanically deflects the hair bundles and opens mechanotransduction channels, which subsequently can produce a receptor potential^3^,^4^. Hair cells are morphologically and functionally polarized to respond to the directional mechanical stimulus: hair cells are activated when the bundle deflects towards the kinocilium^5^. In addition, macular hair cells are arranged in various orientations so that subsets of hair cells respond preferentially to one movement direction^6^,^7^. Behavioural studies that eliminate the otolith organ in fish reveal the functional differences between the three otolith organs: the saccule (S) and lagena (L) are necessary for auditory perception and the utricle (U) is essential for postural equilibrium^2^,^8^,^9^. The mechanisms underlying their functional differentiation, however, remain unclear. Zebrafish are a valuable animal model because the transparency of their embryos and larvae facilitates *in vivo* analyses^8^,^9^. Zebrafish are used in studies that investigate how auditory and vestibular information are distinguished. In the present study, we used zebrafish larvae at 5 days post-fertilization (dpf), because they can sense sound and maintain body posture^8^,^10^,^11^, although neither the L nor the Weberian ossicles have developed yet (Fig. 1a)^12^,^13^ (see Discussions). Focusing on the size difference between the S and U otoliths, we examined whether otolith manipulation could affect sound-evoked microphonic potentials (MPs), which reflect hair cell mechanotransduction responses.

## Results

### S, but not U, hair cells transduce sound into electrical signals

**Figure 1:**
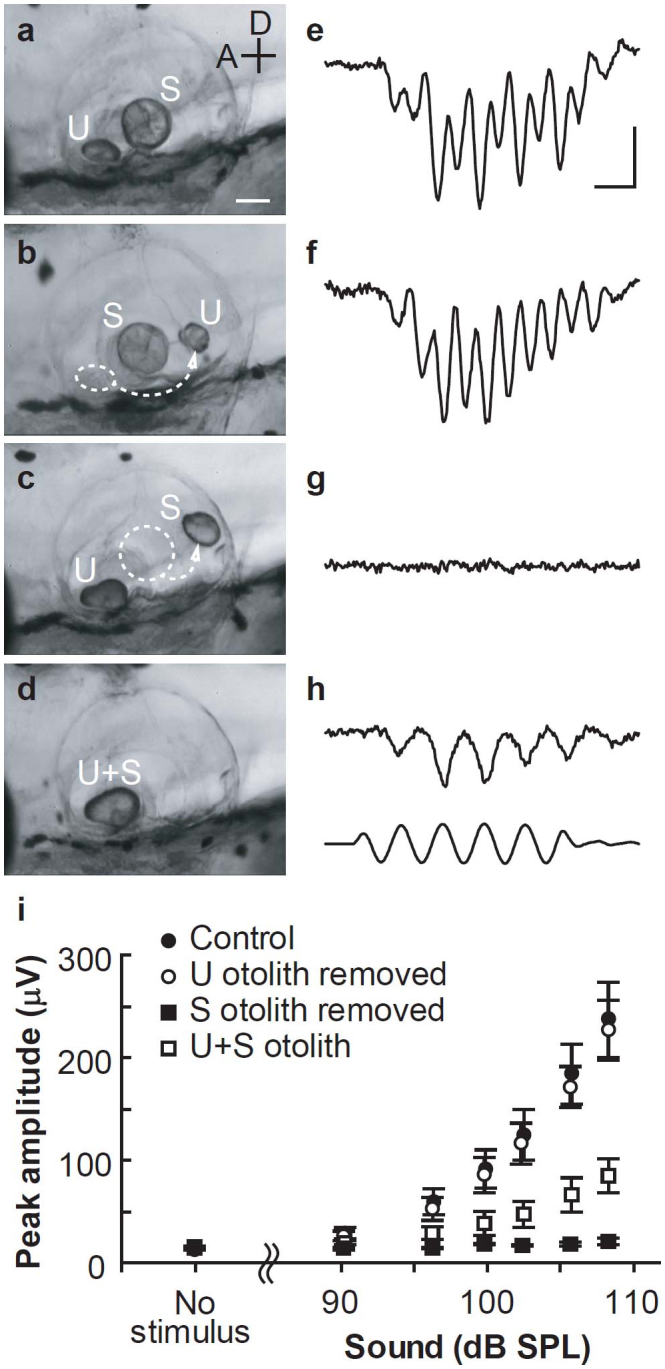
The effect of otolith manipulation on sound-evoked microphonic potentials (MPs). Lateral views of the otic vesicle (OV) (a–d) and MPs (e–h) evoked by sound stimuli [500 Hz, 5 cycles, 108 dB sound pressure level (SPL), h lower waveform] in control (a, e), utricle (U) otolith-removed (b, f), saccule (S) otolith-removed (c, g), and the utricle + saccule (U + S) otolith (d, h) fish. Anterior (A) and dorsal (D) axes and scale bar indicating 50 μm in (a) are also applicable to (b–d). All MP traces (e–h) are averages from 40 consecutive responses. Time (2 ms) and voltage (100 μV) scale bars in (e) are also applicable to (f–h). (i) Peak amplitudes of MPs evoked by various intensities of sound (seven fish in each condition). Error bars denote ± standard error of the mean (s.e.m.).

Similar to previous results, we observed that a sound stimulus (90–108 dB sound pressure level [SPL] at 500 Hz; Fig. 1h lower waveform) elicited negative-going MPs in the otic vesicle (OV), with peaks that occurred at twice the sound frequency (Fig. 1e)^14^,^15^. To determine whether the U or S responded to sound stimulus in larvae, we removed the otolith from each macula in either the U or the S to diminish the magnitude of hair bundle deflection (Fig. 1b, c; see Methods). The sound intensity range used in this study failed to evoke significant MPs in the S-otolith-removed fish (Fig. 1g, i), whereas MP amplitudes in the control and U-otolith-removed fish increased with the sound intensity (Fig. 1f, i). These results indicate that sound stimulus is transduced predominantly by S hair cells in zebrafish larvae.

### Hair cells coupled with a U + S otolith respond to acoustic particle motion

**Figure 2:**
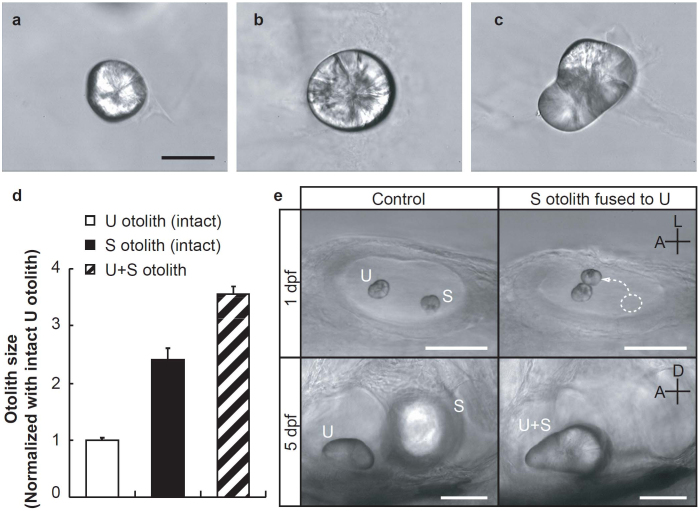
Otolith fusion results in a single large utricle + saccule (U + S) otolith. U (a) and S (b) otoliths isolated from a control larva at 5 days post-fertilization (dpf). A single U + S otolith (c) is produced by fusing the removed S otolith onto the U otolith at 1 dpf; fusion is confirmed at 5 dpf. (d) Relative otolith volume at 5 dpf normalized according to the intact U otolith volume (average of five otoliths) (see Methods). (e) Development of otoliths in control (left) and manipulated fish (right). Upper panels show the dorsal view of the otic vesicle (OV) at 1 dpf, and lower panels show the lateral view of OV at 5 dpf. Anterior (A) and lateral (L) axes and anterior (A) and dorsal (D) axes are applicable to (e) upper and lower left panels, respectively. Scale bars indicate 50 μm in all the figures. Error bars denote standard error of the mean (s.e.m.).

What are the key mechanisms that detect sound stimulus in the S otolith organ? Hair cells coupled with the massive otolith found in the S organ appear to be best suited for sensing acoustic particle motion because a larger otolith with greater inertia more effectively deflects the hair bundles^3^,^4^. Initially, the size of the S and U otoliths were the same at 1 dpf^16^,^17^, but the S otolith showed greater size increases than the U otolith during development (Supplementary Fig. 1). In the 5-dpf larvae, the S otolith volume was 2.5-fold larger than that for the U otolith (Fig. 2a, b, d). We investigated the contribution of otolith size to acoustic sensory transduction by determining whether U hair cells responded to sound stimuli when they were coupled with an enlarged otolith. To make an enlarged otolith in the U macula, we removed the S otolith and fused it to the U otolith at 1 dpf (Fig. 2e upper right). By 5 dpf, the two otoliths had merged into one large otolith, which we termed the U + S otolith (Fig. 1d, 2c, d, e lower right). Surprisingly, sound-evoked MPs were observed in the U + S otolith fish (Fig. 1h). The MP amplitudes in response to 500 Hz at 108 dB SPL were 237 ± 36 μV in controls, 227 ± 30 μV in U-otolith-removed fish, 96 ± 23 μV in U + S otolith fish, and 20 ± 3 μV in S-otolith-removed fish (seven fish were examined in each condition). Amplitudes in the U + S otolith fish were smaller than those observed in controls (*P* = 0.043) or the U-otolith-removed fish (*P* = 0.031), but significantly larger than those in the S-otolith-removed fish (*P* = 0.014). No significant differences were observed in MP amplitudes between the control and U-otolith-removed fish (*P* = 1.0).

### Hair bundle polarity pattern reflects MP frequency

**Figure 3:**
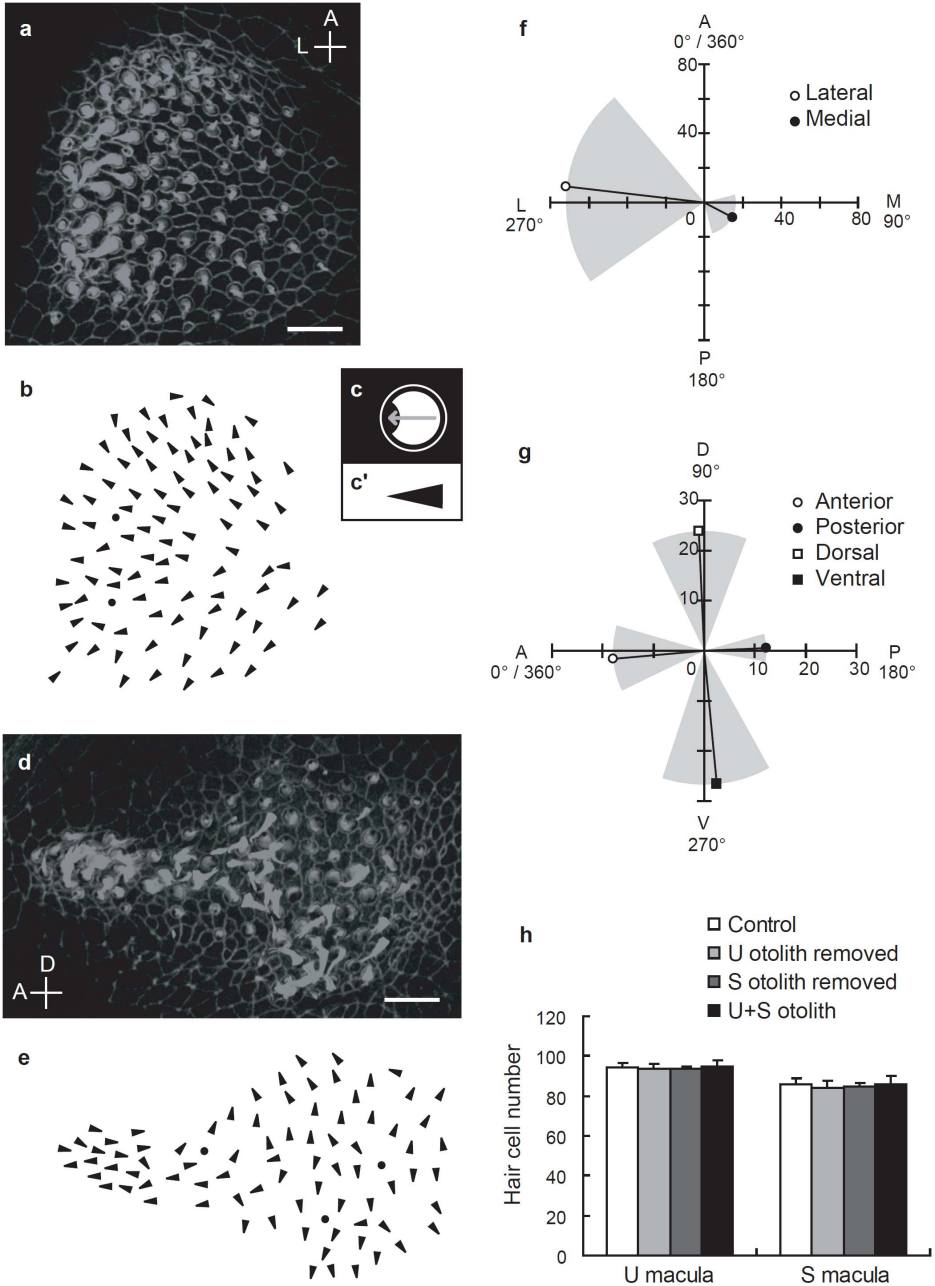
Patterns of hair bundle polarities and hair cell numbers in utricle + saccule (U + S) maculae. Stereocilia and cell boundaries are stained with fluorescent phalloidin in U (a) and S (d) maculae. Since kinocilia are not stained by phalloidin, they appear as dark spots. Hair cell polarities are determined by drawing arrowheads (c′) pointing from the middle of the stereociliary bundles to the middle of the kinocilium (c). (b) and (e) show hair bundle polarities depicted in (a) and (d), respectively. Dots indicate the positions of hair cells with unidentified polarities. (f, g) Quantification of hair bundle polarities and hair cell numbers in control S and U macula (fan-shaped areas [mean (black bars) ± standard deviation (s.d.) (gray areas)] and bar lengths, respectively) (three maculae each) (see Methods). (h) U and S hair cell numbers in control and manipulated-otolith maculae (5 maculae each). Scale bars indicate 10 μm (a, d). Abbreviations in (a, d, f, g) indicate anterior (A), posterior (P), lateral (L), medial (M), dorsal (D), and ventral (V). Error bars denote mean ± standard error of the mean (s.e.m.).

The MP frequency recorded from the U + S otolith fish was identical to the frequency of the sound stimuli (Fig. 1h). This MP frequency differed from the biphasic response recorded from the S macula in controls (Fig. 1e). The response frequency is determined by the hair bundle arrangement in the macula^15^ because mechanotransduction occurs when hair bundles deflect towards the kinocilium^5^. Therefore, we labelled the hair bundles with fluorescent phalloidin to determine the relationship between the MP frequency responses and hair bundle arrangement^18^. In the U macula, the hair bundles were arranged radially from the medial edge to the lateral edge over most of the macula; at the lateral edge, a relatively small number of hair cells were oriented in the opposite direction (Fig. 3a, b, f). In contrast, S hair cells were classified into two anterior groups and two posterior groups. The two anterior groups were arranged in anti-parallel patterns along the anterior-posterior axis, whereas hair bundles in the posterior two groups were arranged in a symmetrical mirror-like pattern along the dorso-ventral axis (Fig. 3d, e, g)^19^. We found that otolith manipulation did not induce any changes in hair cell number (*P* > 0.95; Fig. 3h). These results confirm that the monophasic MPs recorded from U + S otolith larvae were produced by U hair cells coupled with enlarged otoliths, suggesting that enlarged U + S otoliths enable U hair cells to detect acoustic particle motion. Together, our data suggest that otolith size plays a crucial role in acoustic sensory transduction.

## Discussion

On the basis of our knowledge of the adults of most fish species, S otoliths are typically larger than U or L otoliths^20^,^21^, which suggests that fish have improved their sensitivity to the acoustic particle motion by increasing their S otolith size. Otophysian fish, however, including minnows, goldfish, and zebrafish, do not have the large S otolith as adults. Instead, they develop Weberian ossicles that connect the inner ear to the swim bladder, which works as a sound pressure detector that dramatically increases their sensitivity to sound pressure^3^,^4^,^11^. Zebrafish larvae, however, have not yet developed Weberian ossicles^13^, and hence they use a large S otolith to sense the particle motion of the fluid directly^11^. Because the U and S otoliths have similar compositions during development^16^, it is improbable that the displacement of the S otolith nucleus into the U macula at 1 dpf would change the properties of U hair cells. Although it is possible that the differences in the biophysical properties of the otolithic membrane or the hair cell mechanotransduction properties contribute to acoustic particle motion responsiveness in the U and S, the present results indicate that the otolith characteristics largely contribute to the differentiation of auditory and vestibular information in the fish ear. In addition to the otolith organs, the central neural circuits from the S and U are considered important to differentially transmit auditory and vestibular information^3^,^22^,^23^. It would be of interest to determine how the U afferents originating from a U with an enlarged otolith transmit sensory signals.

The mechanisms regulating otolith size and position are of great interest as they are related to vestibular dysfunction. In our study, otoliths removed from maculae, especially the S otoliths, did not grow as large as otoliths that remain in their normal environment (Fig. 1b, c, Supplementary Fig. 1). This indicates that otolith growth occurs in a region-specific manner that is specific to each macula. This finding is consistent with those from previous studies showing that components of the otolithic membrane, and proteins secreted by macular hair cells and supporting cells, are important for otolith development^17^,^24^,^25^,^26^. In addition, fusing the U otolith to the S otolith did not enlarge the otolith size on the S macula (data not shown). This suggests that the growth of the S otolith is tightly regulated during development so that the otolith grows to an appropriate size for acoustic sensory transduction. Previous studies indicate that the genes involved in OV patterning and hair cell formation are differentially expressed along the antero-posterior axis in the developing ear^27^,^28^,^29^. Further studies are required to clarify whether these genes, or other genes and molecules, contribute to macular-specific protein synthesis or secretion processes that in turn contribute to otolith development.

## Methods

### Animals

Adult wild-type zebrafish (*Danio rerio*) obtained from a local supplier (Meito Suien, Japan) were maintained as a breeding colony. Embryos and larvae were reared at 28.5°C and staged according to standard procedures^30^. The experiments described below were performed at 26–28°C. All procedures complied with the guidelines stipulated by the Nagoya University Committee on Animal Research.

### Electrophysiological recordings of MPs

Larvae at 5 dpf were temporally anaesthetised in 0.02% tricaine methanesulfonate (MS-222; Sigma-Aldrich) and then immobilized in 1 mM d-tubocurarine (Sigma-Aldrich) for approximately 10 min. The larvae were securely attached with tungsten pins on a Sylgard-coated glass-bottomed dish filled with extracellular solution containing (in mM) 134 NaCl, 2.9 KCl, 1.2 MgCl_2_, 2.1 CaCl_2_, 10 HEPES, and 10 glucose, at 290 mOsm, adjusted to pH 7.8 with NaOH. The dish was attached to a recording stage (ITS-02, Narishige), using double-sided sticky tape and observed through a 40× water-immersion objective lens (LUMPLFL 40XW, Olympus) equipped on an upright microscope (BX51WI, Olympus). Sinusoidal waveforms for sound stimulation (500 Hz, 5 cycles) were generated by a function generator (Wave Factory 1941, NF Electronic Instruments), amplified by an audio amplifier (PMA-390, Denon), and transmitted into the air through a loudspeaker (101SDVM, Bose) positioned 45 cm lateral to the fish. SPLs were measured using a sound level meter (LA-215, Ono Sokki). Recording micropipettes were made from borosilicate glass capillaries (GD-1.5, Narishige) using a pipette puller (P-97, Sutter Instrument). An MP recording micropipette filled with the abovementioned extracellular solution (resistance, 5–10 MΩ) was inserted into the OV from outside the fish body by using a micromanipulator (MPC-385, Sutter Instrument Co.)^14^,^15^ and was held with a stiff glass rod to reduce vibration during exposure to the applied sound stimuli. Sound-evoked microphonic potentials (MPs) were sampled at 20 kHz using a MultiClamp 700B amplifier (Molecular Devices) and Clampex 10.2 software (Molecular Devices), and were analysed using Clampfit 10.2 software (Molecular Devices).

### Otolith manipulation

Embryos were manually removed from chorions and anaesthetised in 0.02% tricaine methanesulfonate for approximately 3 min and were subsequently held in a Sylgard-coated glass-bottomed dish filled with the abovementioned extracellular solution. A micropipette was inserted into the OV as described above, and the U or S otolith was selectively removed from the macula at 2 dpf. To construct the fused large U + S otolith in the U macula, the S otolith was removed from its macula and positioned adjacent to the U otolith at 1 dpf. Embryos with manipulated otoliths were reared to 5 dpf for further electrophysiological and morphological experiments.

### Otolith size measurement

Otolith size was measured as described previously^31^ with some modifications. Briefly, 5 dpf larvae were anaesthetised in 0.02% tricaine methanesulfonate and embedded in 5% agar, and otoliths were removed from the OVs using a fine tungsten pin. The otoliths were embedded in 5% agar with the macular surface facing downwards. The top-view images (Fig. 2a–c, Supplementary Fig. 1) were captured by a charge-coupled device camera (C2741-79H; Hamamatsu Photonics) mounted on a microscope (BX51WI; Olympus). The otolith was then rotated by 90 degrees, and lateral-view images were captured. The base area and height of the otolith were measured in the captured images using Photoshop 7.0.1 (Adobe). The products of the base area and height were normalized to that of an intact U otolith to calculate the relative volume for each otolith.

### Phalloidin staining and confocal imaging

Five dpf larvae were immunostained using procedures that were described previously^18^,^19^. Larvae were fixed in 4% paraformaldehyde in 0.1 M phosphate-buffered solution (PBS) at 4°C overnight. After permeabilization of the samples with acetone for 10 min at −20°C, hair cell stereocilia were visualized with 1:100 dilution of Alexa Fluor 568 phalloidin (A12380; Invitrogen) in 0.1 M PBS containing 2% Triton X-100.

Coronal and sagittal cryosections of 60-μm thickness were obtained to observe the U and S, respectively. The sections were observed through a 100× oil immersion objective lens on a microscope (BX50WI, Olympus). The fluorescent images were captured at 0.5 μm Z-axis intervals using a confocal laser scanning system (FV300; Olympus) and data acquisition software (Fluoview; Olympus).

### Analysis of hair bundle polarity

The hair bundle polarity was determined by drawing arrowheads pointing from the middle of the phalloidin-stained stereocilia to the kinocilia, which appeared as dark spots (Fig. 3c, c′)^18^,^19^. To quantify hair bundle arrangement in the U, we defined the anterior side as 0°/360°; therefore, the medial, posterior, and lateral directions were expressed as 90°, 180°, and 270°, respectively (in the coronal section, Fig. 3f). Hair cells with hair bundles oriented between 0° and 180° were classified into the medial direction group, and those oriented between 180° and 360° into the lateral direction group (Fig. 3f). Similarly, the anterior side was defined as 0°/360° in S; therefore, the dorsal, posterior, and ventral directions were expressed as 90°, 180°, and 270°, respectively (in the sagittal section, Fig. 3g). S hair cells were divided into two anterior and two posterior groups according to the orientations of their hair bundles: anterior (0°–45° or 315°–360°), dorsal (45°–135°), posterior (135°–225°), and ventral (225°–315°) (Fig. 3g).

### Statistics

All values were expressed as mean ± standard error of the mean, with the exception of the data shown in Figs. 3f and 3g, where the values were expressed as standard deviations. MP amplitudes were statistically analysed using the Steel–Dwass test for nonparametric multiple comparisons. The same results were obtained by the Mann–Whitney test with Bonferroni correction. The number of hair cells was statistically analysed using the Dunnett's test for comparison with control maculae.

## Author Contributions

All the authors contributed to the design of the experiments. M. I. and M. T. developed the experimental procedure. M. I. conducted the experiments and analysed the data, and the results were interpreted by all the authors. All the authors discussed the results and the manuscript was written by M. I. with the help of M. T. and Y. O.

## Supplementary Material

Supplementary Information Supplementary information
