# Supplementary material for: The role of ear stone size in hair cell acoustic sensory transduction
Source: Sci Rep. 2013 Jul 2;3:2114. doi: 10.1038/srep02114 (PMC3698489; doi:10.1038/srep02114)
Supplement: Supplementary Information [file srep02114-s1.pdf]

Supplementary Information

The role of ear stone size in hair cell acoustic sensory transduction

Maya Inoue[1,2], Masashi Tanimoto[1*], & Yoichi Oda[1*]

[1]Division of Biological Science, Graduate School of Science, Nagoya University, Japan

[2]Research Fellow DC of the Japan Society for the Promotion of Science, Japan

*Correspondence should be addressed to either M. Tanimoto or Y. Oda. Email: m-tanimoto@bio.nagoya-u.ac.jp or oda@bio.nagoya-u.ac.jp

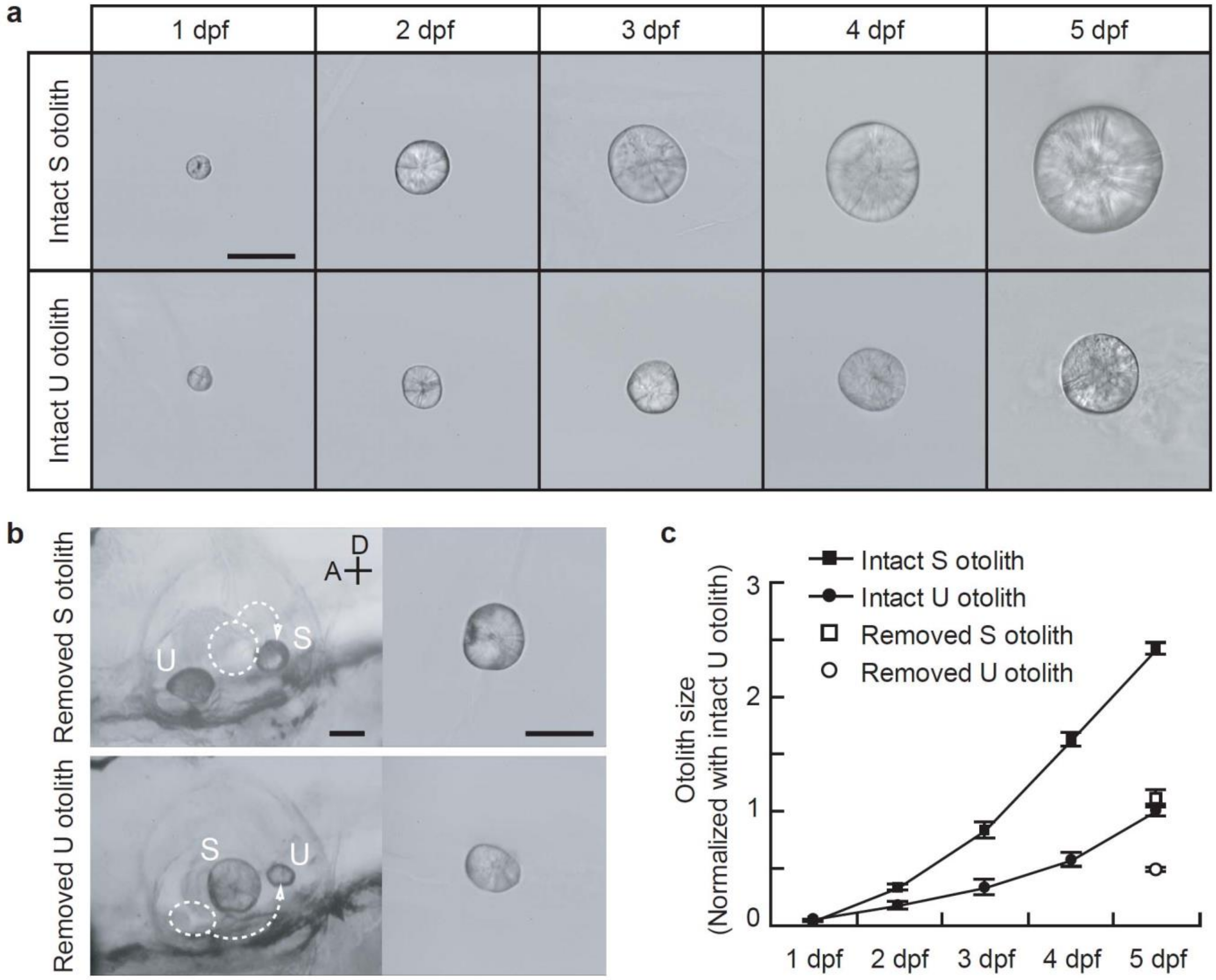

Supplementary Figure S1. Difference in the otolith growth between saccule (S) and utricle (U) and effect of the otolith removal from its macula. (**a**) U and S otoliths isolated from intact larvae at 1 to 5 dpf. (**b**) Manipulated otoliths in S otolith-removed (upper panels) or U otolith-removed fish (lower panels) at 5 dpf. Left panels show lateral views of the otic vesicle (OV), and right panels show isolated otoliths. Otoliths removed from the maculae do not grow as large as normal otoliths do. (**c**) Relative otolith volume normalized according to the volume of intact U otolith at 5 dpf (average of five otoliths). Scale bars represent 50 µm. Abbreviations in (**b**) indicate anterior (A) and dorsal (D). Error bars denote standard error of the mean (s.e.m.).
